# Asylums and Authors

**Published:** 2004

**Authors:** O Somasundaram

**Affiliations:** * Former Director, Institute of Mental Health, Chennai. somasundaram@hotmail.com


*Indian Journal of Psychiatry, 2004, 46(I)29-32*

### Asylums and Authors

**O. Somasundaram***

The role of asylums in the care of the mentally ill has been regarded differently in different times, opinions held by a person even being reversed totally with the passing of time! In the early part of asylum history the majority of alienists felt that "all forms of madness required institutional care and treatment and the sooner those displaying signs of mental imbalance were removed from domestic to asylum care, the better for all concerned, with better chances of an ultimate recovery" (Scull et all, 1999).

The range of indications supposedly making one an asylum candidate widened to include, in the words of John 'non-restraint. Connolly of Hanwell asylum: "young men of gross habits, love of drink, dishonest or irregular conduct; women of ungovernable temper, sullen, wayward, malicious, defying domestic control and wanting for restraint over the passions."

The evolution of mental health legislation in Britain is ably covered by Kathleen Jones(1972) and the corresponding Indian scene by Somasundaram(1987).

The legalism engendered by the 1890 Lunacy Act and the subsequent 'custodial' approach led to the stagnation and decline in standards of care and treatment in all parts of the British Empire, including India.

This has not improved much in independent India after liberation. Our current conditions are detailed exhaustively in the National Human Rights Commission's 2000 Report (NHRC 2000.)

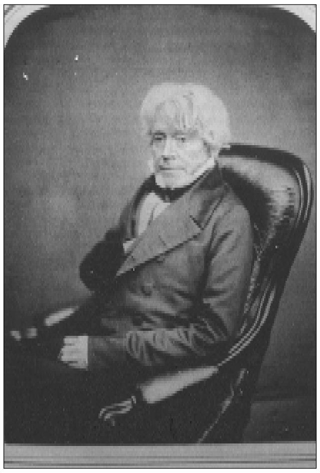

(John Conolly as an old man: February 1866, a few days before his death)

**Charles Reade's "Hard Cash" :**

Charles Reade, a popular Victorian novelist remembered for his "The Cloister And The Hearth" was known for painstaking research before writing, in this case on the prevailing conditions and practices of the time with regard to mental illness and it's care and society.

"Hard Cash" published in 1868 (long out of print but fortunately easily obtained from the Internet) is the story of two families, with an ex sea-captain suffering from a long mental illness following an apoplexy and kept in an asylum after being certified. Dr. Wycherly ( the fictional counterpart of John Conolly) is a self professed "benevolent gentleman doctor" involved in the fraudulent certification and incarceration of normal individuals in his asylum, where "there are no tortures, no handcuffs nor leg-locks, no brutality". The doctor, "blinded by self-interest" is "apt to perceive insanity wherever he looked" with "a tendency to found facts on theories instead of theories on facts".

In the pivotal courtroom scenes where Dr. Wycherly is put on the witness stand and questioned (as Conolly was in reality), he's forced to admit(as Conolly did) that he received a substantial income from his role in getting asylum keepers a steady stream of patients/inmates.

There is also a nice description of the asylum being readied for an official inspection with much cleaning and washing, patients primed to report "satisfaction"; difficult inmates, chains and restraints being hidden away. The inspectors don't want to take much trouble in their inspection and of course ready to take the asylum keepers' word against that of any patient.

**Samuel Beckett's "Murphy" :**

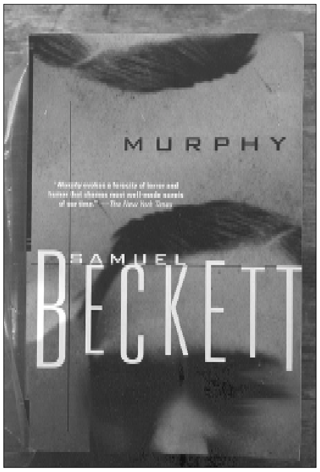

1969 Nobel winner for Literature, Beckett created this in 1938 - his first novel, the fore-runner of his brilliant and highly individual works. Beckett's biography is interesting from a psychiatric point of view as he suffered from repeated, protracted bouts of depression and anxiety. On his old friend Dr. Thompson's advice he sought out Dr. Wilfred Bion at the Tavistock Clinic at London for supportive psychotherapy twice weekly for almost two years. After his friend Dr. Thompson moved in 1934 to London to train in psychiatry at the Bethlem Royal and the Maudsley Hospital, Beckett regularly visited him at Bethlem, walking the grounds, visiting the wards and

*Former Director, Institute of Mental Health, Chennai. Email : somasundaram@hotmail.com playing chess with him (Farell, 1987.)

Beckett depicts and describes Bethlem Royal as Magdalen Mental Mercyseat —MMM — where Murphy gets to work in an "overheated atmosphere stinking of paraldehyde and truant sphincters" among the patients of Skinner's House - "melancholics motionless and brooding, holding their heads or their bellies according to type" with "paranoids feverishly covering paper with complaints against their treatment or verbatim reports of their inner voices" and "a hebephrenic playing the piano, a hypomanic teaching slosh to a Korsakow's syndrome and a schizoid petrified in a toppling attitude, left hand extended holding a cigarette and the right pointing upward, quivering and rigid."

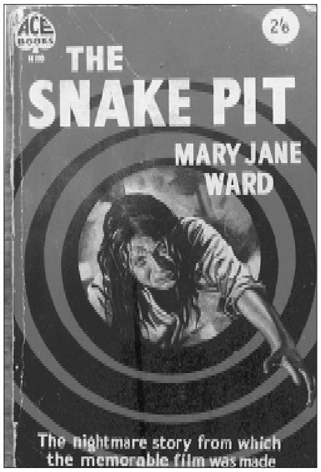

**Mary Jane Ward's "The Snake Pit" :**

Extremely pitiable and appallingly poor conditions prevailed in the State Mental Hospitals in mid-20th century American states — "The Shame of The States" in the words of Albert Deutsch the well-known American psycho-historian.

The protagonist of Ward's evocatively written 1946 novel, Virginia ( portrayed by Olivia De Havilland in the movie version) takes us along on her long journey to recovery from mental illness as a patient admitted in a sanatorium, allowing us a very life-like first person view of what it feels like.

M.J. Ward based the novel on her own personal experiences — she underwent psychiatric treatment and hospitalisation for her own psychotic breakdown. Some illuminating excerpts from the book are now given here.

**Why the title "The Snake pit" :**

"Long ago they lowered insane persons into snake pits; they thought that an experience that might drive a sane person out of his wits might send an insane person back to sanity."

***An ECT Scene:***

"She was taken down the hall and the moment she saw the room she knew she had been shocked previously and that she did not care for another helping….the room smelled like her old egg-beater and there was a dull red glass eye in the wall….there was a high table like an operating table and she knew she was supposed to get up on it….they put a wedge under her back. It was most uncomfortable….the woman was applying a sort of cold paste to her temples….now putting clamps on her head, on the paste-smeared temples….another nurse-garbed woman and she leaned on her feet as if she might rise up from the table and strike the ceiling….hands tied down, legs tied down. She opened her mouth to call for a lawyer and the silly woman thrust a gag into it….soon it would be over."

***At The Hospital Toilet*:**

"there were four booths. The women stood and waited their turn….pushing was done quietly….none of the booths had a door….the toilet had no wooden seat….and no toilet paper….she found out Miss Hart was the dispenser. If you required paper you asked her for it in advance and she doled it out to you. She was the judge of how much you needed. It was a curious and humiliating procedure."

***Juniper Hill Hospital:***

"There wasn't enough of anything at Juniper Hill. Not enough doctors, not enough nurses, not enough toilet paper, not enough food and not enough covers for cold nights. When the laundry didn't get to the wards in time there were not enough sheets and not enough pillowcases. As Virginia knew, there were not enough beds."

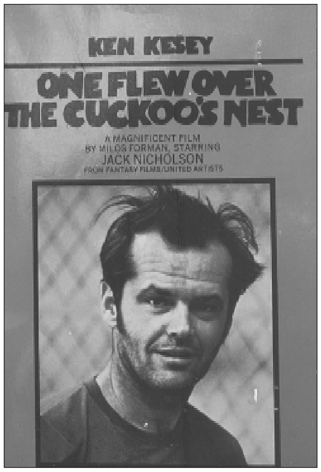

**Ken Kesey's "One Flew Over The Cuckoo's Nest":**

This outstanding novel on a State Mental Hospital written in 1962 was turned into a famous Academy Award winning 1969 film of the same name starring Jack Nicholson.

Kesey, during his student days, had taken part as a "guinea-pig" in "psychedelic" experiments involving marijuana, peyote, psilocybin etc. as part of research. He fell violently in love with the mind-altering effects of drugs, which he wanted others to experience too! He also later served a prison sentence of five months for illicit drug possession.

This is the man almost single-handedly responsible for the drug-induced "psychedelic" era of the early sixties through half the seventies, millions of youth influenced by their icons of the music and movie world, who in turn were led by Ken Kesey into mesmerising, "hallucinogenic trips", starting by being introduced at his wild parties to "LSD-laced punch"

and other assorted drugs.

This book exerted the maximum influence in bringing ECT into the limelight with much disrepute, highlighting and exaggerating the procedure and it's effects, horrifying and incensing droves of people who demanded for and brought ECT under strict legislation and control. Utah in 1967 was the first American state to bring such legislation and by 1983. 26 states had passed some kind of statute, six others issued administrative regulations and one state was operating under Federal Court orders. In some jurisdictions, ECT was actually banned.

So for about a quarter-century, public alarm and widespread media coverage ensured the virtual disappearance of ECT from even University training programs, leaving the younger generations of psychiatrist trained after the sixties unfamiliar with ECT.

Kesey's novel deals with the treatment meted out to a person suffering from a personality disorder in a State Mental Hospital who comes to clash with the "Big Nurse" ruling the institution on behalf of the all-powerful administrators. Kesey's experiences with psychiatric patients dates back to his work as an orderly at a local Veterans Administration hospital.

The classification of the patients as "Acutes" - patients considered "sick enough to be fixed" and the more severe "Chronics" — patients who are in for good, further subdivided into "Walkers" "Wheelers" and "Vegetables" is self-explanatory.

The most shocking scene is probably the description of EST — Electro Shock Therapy:

"…that filthy brain-murdering room that the black boys (the orderlies) call the Shock Shop…"

"…They brought him back to the ward two weeks later, bald and the front of his face an oily purple bruise and two little button-sized plugs stitched one above each eye. You can see by his eyes how they burned him out over there; his eyes are all smoked-up and grey and deserted inside like blown fuses. All day now he won't do a thing…."

"…They gave McMurphy three more treatments that week. As quick as he started coming out of one, Miss Ratched would arrive with the doctor and they would ask him if he felt like he was ready for another…he'd swell up, aware that every one of those faces on ............"

Disturbed had turned towards him…he'd tell the nurse he regretted that he had but one life to give…he'd stand up and take a couple of bows…while the nurse led the doctor into the station to phone the Main Building and authorise another treatment."

The "Big Nurse" uses the "administrative" EST to "soften" behaviours. When this is not successful with McMurphy she proceeds to the ultimate solution — bilateral lobotomy:

"I was suggesting that we consider an operation — very simple really. And we've had a history of past successes eliminating aggressive tendencies in certain hostile cases."

After McMurphy's been operated upon and returned to the ward his friend "Chief Broom" cannot bear the ignominious sight of his lively and exuberant friend's now mindless body lying vegetable-like, maybe for even decades, as an "example" of the folly of rebelling against the all-powerful Administration till the vestiges of life left drained away. He knows that McMurphy would never have allowed this if he was capable of conscious action, so the "Chief" (he's of Red-Indian descent) takes McMurphy's life by suffocation.

In the various author's works quoted from above, different aspects outlined are:

- Charles Reade's obsession with highlighting the illegal commitment of normal people, especially to private asylums.
- Beckett's book deals with the fairly satisfactory conditions prevailing at the Bethlem Royal (the basis for his fictional hospital.) Bethlem should bring back plenty of nostalgic associations for the many Indian colleagues trained there in the 1950s and 1960s.
- M.J. Ward's "The Snake Pit" takes us through the nightmarish conditions and ill-treatment afforded to patients in "The Shame Of The States" hospitals in the 1940s.
- Kesey's "One Flew Over The Cuckoo's Nest" was one of the main vehicles wherein the horrifying descriptions of indiscriminate "punitive" ECT and lobotomy made an enraged American public demand for strong legislation and control of such procedures.

**Implications for Indian Psychiatry:**

Following the Mental Health Act 1987, many private psychiatric services are being licensed. The monitoring of admission to and administration of these should be strictly done to avoid the "mad-doctoring trade", in the words of Andrew Scull.

The physical environment of places offering psychiatric services should be conducive to providing humane treatment with a therapeutic atmosphere. Outmoded procedures like “straight” ECT and psycho-surgery should be avoided.

### REFERENCES

Beckett, Samuel (1938): Murphy. Grove Press, New York.

Farell, M.(1987): Beckett’s Bedlam. The Maudsley And Bethlem Gazette Vol.34 Nos. 1-2, pp 24-26.

Kesey, Ken (1962): One Flew Over The Cuckoo’s Nest. Methuen, London.

Jones, Kathleen (1972): A History Of The Mental Health Services. Routledge and Kegan Paul, London.

Jones, Kathleen (1993): Asylums And After. The Athlone Press, London and Atlantic Highlands New Jersey.

National Human Rights Commission (2000): Quality Assurance In Mental Health. NHRC, New Delhi.

Reade, Charles (1868): Hard Cash.Project Gutenberg e-text from Internet.

Scull A., Mackenzie C., Hervey N.(1999): Masters Of Bedlam. Princeton University Press, Princeton New Jersey.

Somasundaram O.(1987): Indian Lunacy Act 1912 - The Historical Background. Indian Journal Of Psychiatry Vol.29(1) pp 3-14.

Ward, Mary Jane (1946): The Snake Pit. Cassell, London.

